# Flavin Biosynthesis Enhances Extracellular Electron Transfer in Bioengineered *Escherichia coli*

**DOI:** 10.1002/advs.202412230

**Published:** 2025-11-01

**Authors:** Mohammed Mouhib, Melania Reggente, Hanxuan Wang, Charlotte Roullier, Ardemis A. Boghossian

**Affiliations:** ^1^ Ecole Polytechnique Fédérale de Lausanne (EPFL) Lausanne 1015 Switzerland

**Keywords:** bioelectrochemistry, extracellular electron transfer, microbial fuel cell, redox mediator, synthetic biology

## Abstract

Advancements in bioengineering have unlocked new microbial electrochemical applications in energy, sensing, remediation, and synthesis. Key to realizing these technologies is the engineering of conduits in metabolically versatile microbes like *Escherichia coli* to enable efficient charge exchange with the electrode. Inspired by mechanisms found in natural exogelectrogens, previous studies have largely focused on introducing conduits based on the metal‐reducing (Mtr) pathway in *Shewanella oneidensis* MR‐1. This study explores the concomitant expression of flavin secretion pathways for mediated charge transfer to complement the direct charge transfer from the bioengineered Mtr pathway. The engineered strains show a 3‐fold increase in the total secretion of flavin mononucleotide (FMN) and riboflavin compared to a state‐of‐the‐art Mtr‐expressing strain lacking flavin overexpression. The concomitant flavin secretion further contributes up to a ≈3.4‐ and ≈1.5‐fold increase in current compared to unmodified cells and the previous Mtr‐expressing cells, respectively, with the greatest currents achieved for the strain favoring riboflavin secretion over FMN secretion. The introduction of flavin biosynthesis genes to Mtr‐expressing strains thus reveals a distinct, yet complementary, EET mechanism for robust and multi‐modal microbial applications.

## Graphical abstract

Microbial electronics are promising for energy, sensing, environmental, and synthesis applications. *E. coli* are engineered with extracellular electron transfer (EET) pathways from a microbe that naturally produces current to enable bioelectronics based on *E. coli*.

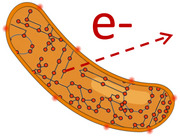

## 1 Introduction

Microbes offer a largely untapped potential for developing sustainable electrochemical technologies for energy,^[^ ^1^ ^]^ sensing,^[^ ^2^, ^3^, ^4^ ^]^ environmental,^[^ ^3^, ^4^, ^5^ ^]^ and synthesis^[^ ^6^, ^7^, ^8^ ^]^ applications. These technologies rely on extracellular electron transfer (EET), which enables the exchange of energy and information between microbes and electrodes. Such technologies benefit from microbes that combine efficient EET with application‐specific metabolic abilities, such as the ability to catabolize certain metabolites, react to environmental stimuli, deactivate pollutants, or produce specific chemicals.

Although several microbes naturally possess EET abilities,^[^ ^9^ ^]^ *Shewanella oneidensis* MR‐1 (S. oneidensis MR‐1) benefits from the extensive study and application of its EET mechanisms.^[^ ^1^, ^10^ ^]^ In *S. oneidensis* MR‐1, electrons are transferred to extracellular electron acceptors through the Mtr pathway as well as through soluble, cytochrome‐based flavins.^[^ ^10^, ^11^ ^]^ The Mtr pathway consists of an inner‐membrane quinol oxidase, periplasmic cytochromes, and the outer membrane‐spanning Mtr complex.^[^ ^10^, ^11^ ^]^ Throughout the pathway, electrons are transferred through covalently bound heme cofactors in c‐type cytochromes. In addition to direct EET from the Mtr pathway, *S. oneidensis* MR‐1 are also capable of indirect EET through the secretion of soluble flavins.^[^ ^12^, ^13^, ^14^ ^]^ In fact, c.a. 70% of the EET in *S. oneidensis* MR‐1 has been attributed to the secretion of flavins, with riboflavin as the most prevalent.^[^ ^12^ ^]^

While these mechanisms enable EET in native exoelectrogens like *S*. *oneidensis* MR‐1, native exoelectrogens often lack the substrate promiscuity and genetic amenability to realize most applications. *Escherichia coli (E. coli)* have thus emerged as a promising alternative to native exoelectrogens. Unlike most native exoelectrogens, *E. coli* can metabolize a broad range of substrates. This versatility allows these microbes to act as metabolic‐driven sensors and sequesters of toxic compounds for sensing and bioremediation applications, respectively. The substrates can also serve as fuels in a microbial fuel cell (MFC), favoring *E. coli* over native exoelectrogens in pure‐culture MFCs that are powered by non‐native substrates.^[^ ^15^ ^]^ Importantly, *E. coli* have a highly developed synthetic biology toolbox. This toolbox diversifies the range of substrates and products that *E. coli* can consume and produce, respectively, through genetic engineering. This diversification is especially advantageous for electrosynthesis applications that seek to convert a limited selection of substrates to high‐value products.

Despite their genetic and metabolic versatility, *E. coli* show limited EET compared to native exoelectrogens. Recent efforts have therefore focused on engineering exogenous EET pathways in *E. coli*.^[^ ^4^, ^6^, ^15^, ^16^, ^17^, ^18^, ^19^, ^20^, ^21^, ^22^ ^]^ The past two decades have seen significant advancements in expressing the Mtr pathways of *S. oneidensis* MR‐1 in *E. coli*, from the expression of MtrA^[^ ^22^ ^]^ to the Expression of the MtrCAB complex in the outer membrane^[^ ^16^ ^]^ to the co‐expression of the inner‐membrane cytochrome CymA with the MtrCAB complex^[^ ^18^ ^]^ and, finally, the expression of the entire pathway, including both the membrane‐associated and periplasmic cytochromes.^[^ ^15^ ^]^ The implementation of these direct EET mechanisms in *E. coli* has already led to new applications in the electro‐biosynthesis of chemicals and biosensing.^[^ ^4^, ^6^, ^17^, ^20^, ^23^ ^]^

Beyond direct electron transfer, the EET in engineered *E. coli* can be further improved with mediators.^[^ ^6^, ^17^, ^18^, ^21^, ^23^ ^]^ Most common lab strains of *E. coli* show limited biofilm formation,^[^ ^24^ ^]^ limiting the direct electron transfer to the few cells that are non‐specifically adsorbed onto the electrode. Although exogenous mediators may enhance EET through indirect EET, they often entail additional costs and technical challenges, such as toxicity and instability.^[^ ^25^ ^]^ The introduction of biosynthesizable mediators, such as secreted phenazine‐1‐carboxylic acid from bioengineered *E. coli*,^[^ ^26^ ^]^ thus offers a promising approach to overcoming these challenges.

Biosynthesizable flavins, in particular, play a crucial role in enhancing EET as effective redox mediators. For example, *S. oneidensis* MR‐1 has been co‐cultured with flavin‐secreting bacteria or genetically modified with a flavin biosynthesis module to increase flavin production and enhance electrochemical performance.^[^ ^27^, ^28^, ^29^ ^]^ The heterologous expression of a flavin biosynthesis pathway from *Bacillus subtilis* in *S. oneidensis* MR‐1 was also shown to enhance EET in a MFC, leading to a 13‐fold increase in the maximum power output.^[^ ^29^ ^]^ The biosynthesis of flavins has also been demonstrated in *E. coli* for riboflavin production.^[^ ^30^ ^]^ While *E. coli* natively encodes the genes necessary for flavin biosynthesis, their overexpression, as well as the introduction of exogenous pathways from strains such as *Bacillus subtilis*, provide a promising avenue for increasing the secreted flavin concentrations.^[^ ^30^ ^]^

**Figure 1:**
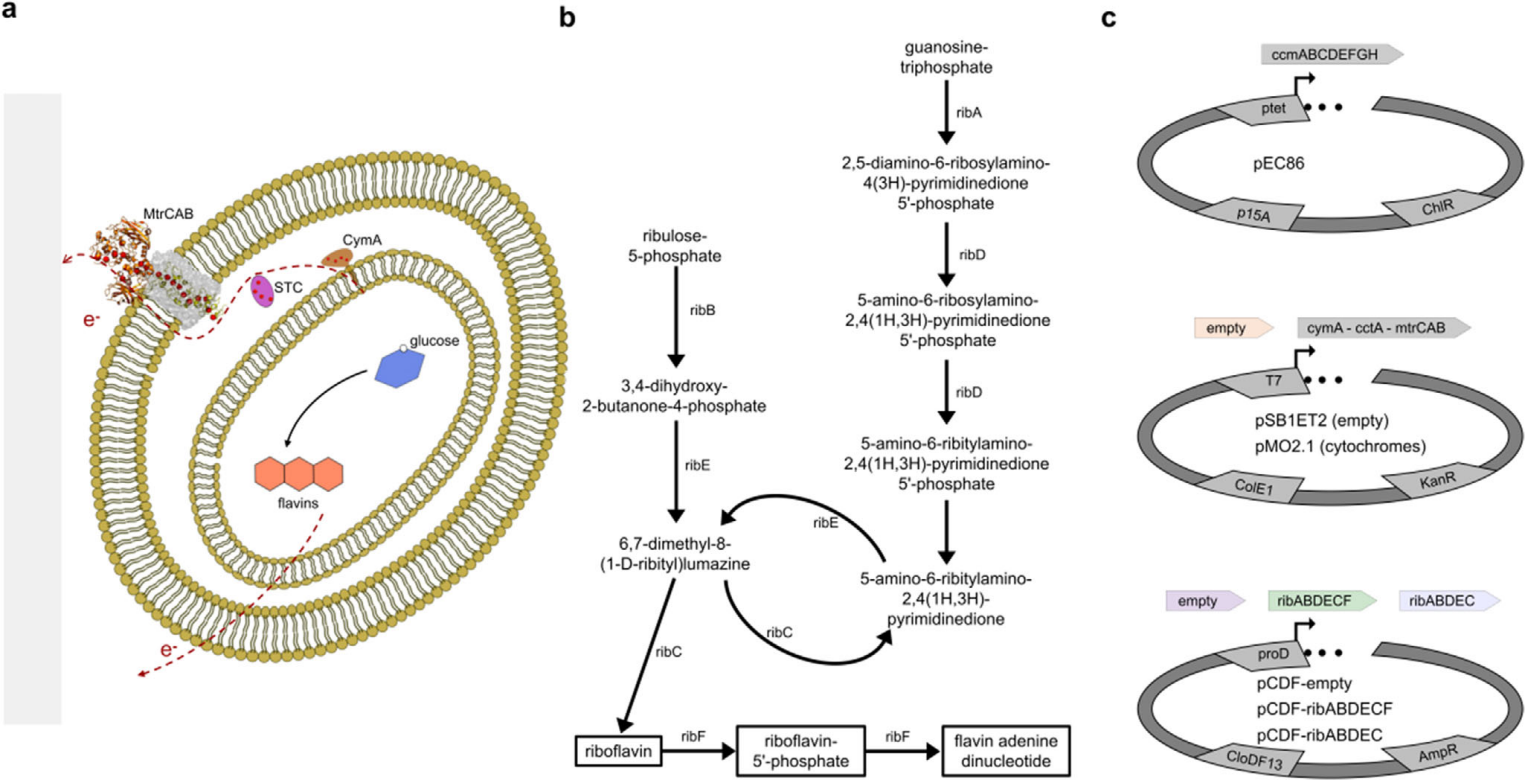
Co‐expression of the Mtr and flavin biosynthesis pathways. a) Schematic of an *E. coli* cell expressing both a flavin biosynthesis pathway and the Mtr pathway across the inner membrane, periplasm, and outer membrane. The schematic is not drawn to scale; the substrates, proteins, membranes, and periplasmic space have been exaggerated to emphasize the related mechanisms. b) Overview of the flavin biosynthesis pathway expressed in this study. Genes encoding enzymes catalyzing a given reaction are denoted next to the arrows between the metabolites. c) Illustration of plasmids used in this study, including the promoters (ptet, T7, proD), origins of replication (p15A, ColE1, CloDF13), and antibiotic resistance genes (ChlR–chloramphenicol, KanR–kanamycin, AmpR–ampicillin). Expressed genes of interest are illustrated by arrows above the corresponding plasmids.

Given the importance of flavins in *S. oneidensis* MR‐1,^[^ ^12^ ^]^ this study aims to explore the biosynthesis of riboflavin and flavin mononucleotide (FMN) in *E. coli* that have been previously bioengineered to express the Mtr pathway (**Figure** **1**a). Beyond enhancing the EET, the flavin biosynthesis introduces an orthogonal mechanism for EET that is based on indirect electron transfer, complementing existing bioengineered pathways based on direct EET. Such orthogonality enables multi‐modal applications in sensing and the electrosynthesis of diverse products based on distinct electron transfer pathways.

## 2 Results and Discussion

The bioengineering strategy herein leverages *E. coli*′s endogenous biosynthesis of riboflavin and FMN (Figure 1b).^[^ ^30^ ^]^ Collectively, the GTP cyclohydrolase II (*ribA*), 3,4‐dihydroxy‐2‐butanone 4‐phosphate synthase (*ribB*), fused diaminohydroxyphosphoribosylaminopyrimidine deaminase / 5‐amino‐6‐(5‐phosphoribosylamino)uracil reductase (*ribD*), 6,7‐dimethyl‐8‐ribityllumazine synthase (*ribE*), g and riboflavin synthase (*ribC*) enable the biosynthesis of riboflavin from guanosine triphosphate and ribulose‐5 phosphate. The subsequent conversions of riboflavin to FMN and flavin adenine dinucleotide are catalyzed by a bifunctional riboflavin kinase / FMN adenylyltransferase.^[^ ^30^, ^31^ ^]^ The encoding genes, *ribABDECF*, are not naturally located in one operon on the chromosome.^[^ ^31^ ^]^ This strategy thus relies on a synthetic operon for the expression of either *ribABDEC* (pCDF‐ribABDEC) to accumulate riboflavin or *ribABDECF* (pCDF‐ribABDECF) to accumulate FMN under the control of a strong constitutive promoter.^[^ ^32^ ^]^ In addition to a plasmid dedicated to flavin biosynthesis, the *E. coli* were also transformed with an additional plasmid for the expression of the Mtr pathway (pMO2.1)^[^ ^15^ ^]^ and a plasmid for expressing the Ccm machinery (pEC86)^[^ ^33^ ^]^ necessary for the post‐translational modification of c‐type cytochromes. These four plasmids, along with negative control plasmids for the Mtr pathway expression (pSB1ET2) and flavin biosynthesis (pCDF‐empty), were transformed in different combinations to create a total of five modified strains (Figure 1c; **Table** **1**).

**Table 1:** Summary of transformed plasmids corresponding to each strain. Each strain comprises three plasmids: a plasmid for the expression of the Ccm pathway (Ccm plasmid), a plasmid for the expression of the apo proteins in the STC, CymA, and MtrCAB pathway (Mtr plasmid), and a plasmid for the overexpression of the flavin biosynthesis genes (Flavin plasmid). Plasmids denoted with (+) correspond to positive control vectors comprising the backbone with the gene of interest, while plasmids denoted with (−) correspond to negative control vectors comprising the backbone without the gene.

Ccm Plasmid	Mtr Plasmid	Flavin Plasmid	Strain Name
pEC86 (+)	pSB1ET2 (−)	pCDF‐empty (−)	empty
pEC86 (+)	pSB1ET2 (−)	pCDF‐ribABDEC (+)	*rib‐dF‐dCyt*
pEC86 (+)	pMO2.1 (+)	pCDF‐empty (−)	*cymstcmtr*
pEC86 (+)	pMO2.1 (+)	pCDF‐ribABDECF (+)	*rib*
pEC86 (+)	pMO2.1 (+)	pCDF‐ribABDEC (+)	*rib‐dF*

**Figure 2:**
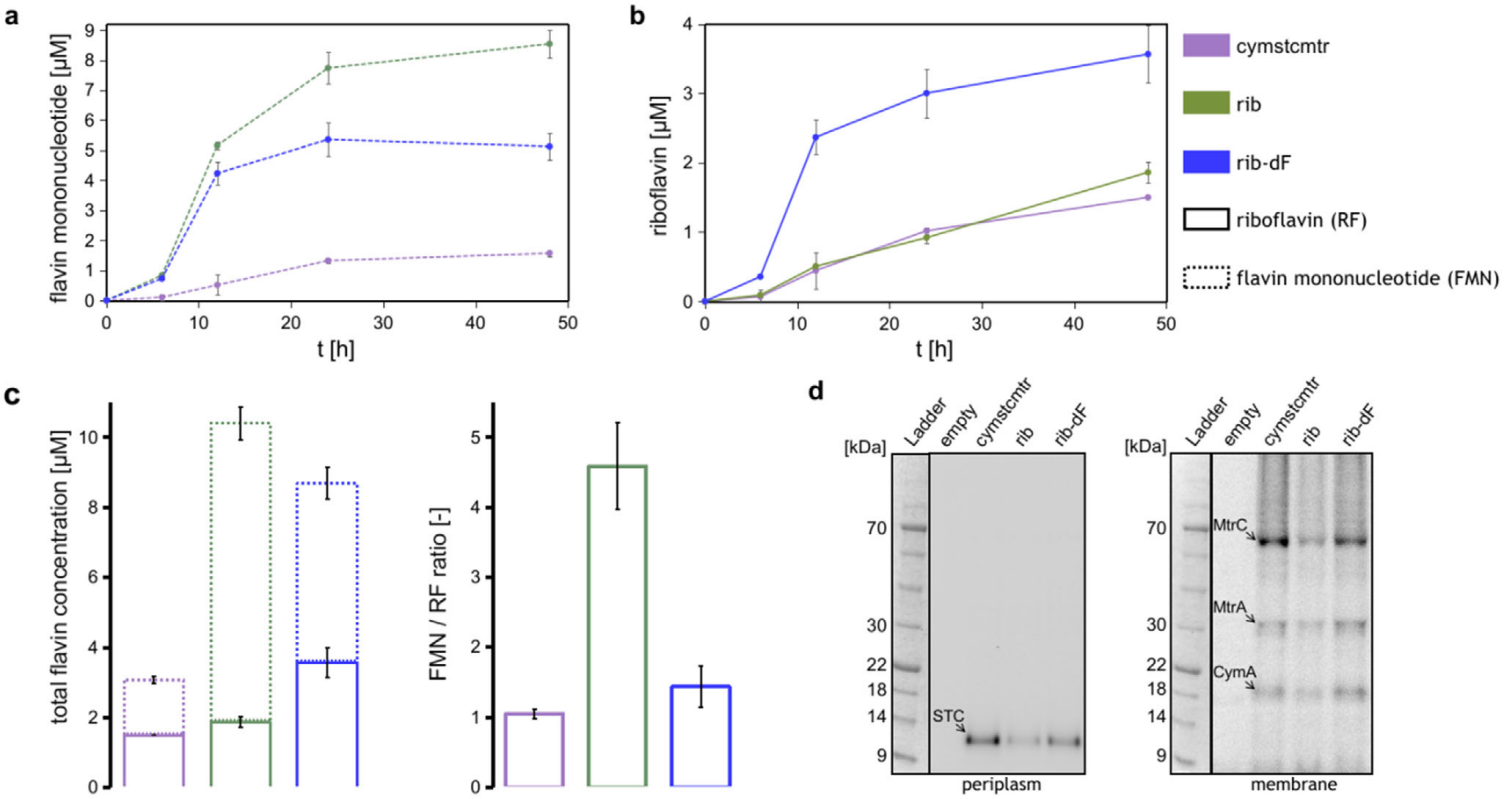
Flavin secretion and cytochrome expression. The concentrations of a) FMN and b) riboflavin over time during aerobic growth in glucose‐supplemented M9 medium. c) Total flavin concentration (left) and the ratio of FMN to riboflavin (FMN/RF) (right) after 48 h of aerobic growth. d) SDS‐PAGE gels (4%–20%, MOPS‐SDS buffer, 30 µg of protein per lane) loaded with periplasmic (left) and membrane (right) protein extracts. The gels were stained for hemes using an enhanced chemiluminescence substrate. Mean values of 3 independent biological replicates are shown, with error bars representing 1 standard deviation.

To evaluate the Mtr pathway expression and flavin secretion, the strains were grown aerobically in a minimal medium with glucose as the carbon source. Growth (Figure S1, Supporting Information) and flavin concentration measurements (**Figure** **2**a,b) were taken for MtrCAB‐expressing strains with no flavin biosynthesis module (cymstcmtr), with co‐expressed *ribABDECF* genes (rib), and with co‐expressed *ribABDEC* genes (rib‐dF). While all strains achieve comparable optical densities, secreting both riboflavin and FMN over the course of 48 h, the strains expressing either one of the flavin modules secrete significantly higher amounts of FMN (Figure 2a). In addition, the rib‐dF strain also shows a significant increase in riboflavin concentration throughout the measurement (Figure 2b). After 48 h, the rib strain accumulates the highest total flavin concentration (Figure 2c) at 10.4 ± 0.61 µM, representing a 20% increase over the rib‐dF strain (8.7 ± 0.85 µM) and a 3‐fold increase over the cymstcmtr strain (3.07 ± 0.1 µM). Most of the increase is attributed to FMN secretion, with only a 24% increase in the riboflavin secreted from the rib strain over the cymstcmtr strain. By contrast, the rib‐dF strain shows a larger relative contribution from riboflavin, achieving a 2.4‐ and 3.3‐fold increase in the riboflavin and FMN concentrations, respectively, compared to the cymstcmtr strain. This difference in the FMN to riboflavin ratios of the rib and rib‐dF strains is expected, since the deletion of the *ribF* gene from the *ribABDECF* operon reduces the amount of the riboflavin kinase that is available for the phosphorylation of riboflavin to FMN. Though flavin synthesis was also confirmed under anaerobic conditions (Figure S2, Supporting Information), the flavin concentrations were significantly diminished compared to those produced under aerobic conditions.

The effect of the flavin overexpression on the localization and post‐translational modification of Mtr pathway cytochromes was studied with SDS‐PAGE (Figure 2d). Subcellular fractions of modified *E. coli* and enhanced chemiluminescence (ECL) staining were used to identify full‐length c‐type cytochromes in the cell lysates. All the strains express the small tetraheme cytochrome STC (*cctA* gene product) in the periplasm as well as the inner‐membrane cytochrome CymA and components of the Mtr complex (MtrA, MtrC) in the membrane fraction. Total protein staining was used to keep the protein concentrations in the periplasmic and membrane extracts constant between the different strains (Figure S3, Supporting Information). At equivalent loadings, the cytochrome concentrations appear higher in the absence of a flavin‐secretion pathway (cymstcmtr) compared to the flavin‐secreting rib and rib‐dF strains (Figure 2d; Figure S4, Supporting Information). This trend is also consistent with that of the flavin secretion strains, with the rib‐dF strain, which shows a lower total flavin secretion (Figure 2c), showing increased cytochrome expression compared to the rib strain.

**Figure 3:**
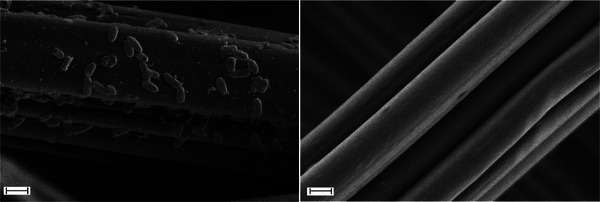
EET characterization with chronoamperometry. a) Chronoamperometric measurements under anaerobic conditions in glucose‐supplemented M9 medium. Measurements were taken at a potential of +0.2 V vs. Ag/AgCl, and current values were recorded every 30 s. Mean currents of three independent measurements are shown (two measurements for the empty vector control), with the shaded area representing one standard deviation. b) The OD_600_ in solution measured after chronoamperometry. The horizontal dashed line corresponds to the starting OD_600_ of 0.6. Bars represent mean values of three measurements, with the error bars corresponding to one standard deviation.

**Figure 4:**
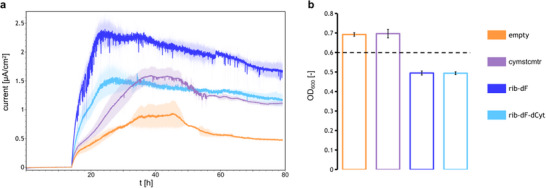
SEM micrographs of a graphite felt working electrode (left) after chronoamperometry measurements with *E. coli* and (right) of a blank graphite felt sample. Images are shown at 10000x magnification with scale bars representing 2 µm.

The effect of the flavin biosynthesis on EET was characterized through chronoamperometry (Figure 4; Figure S5a, Supporting Information). The chronoamperometry measurements were taken in M9 medium under anaerobic conditions with glucose as the sole carbon source and a graphite felt working electrode as the electron acceptor (+0.2 V vs. Ag/AgCl). Unlike previous studies that relied on fixation with a dialysis membrane to induce biofilm formation on graphite,^[^ ^34^ ^]^ these measurements were taken in free solution, relying on the cells’ inherent ability to immobilize on the electrode. The cells are based on BL21‐derived strains that show defective cellulose biosynthesis, flagella formation, and *lsr* quorom sensing, characteristics that are associated with limited biofilm formation.^[^ ^35^, ^36^, ^37^ ^]^ Though these cells do not form thick biofilms on the electrode under these conditions, scanning electron microscopy (SEM) images nonetheless confirm the non‐specific attachment of individual cells and cell agglomerates (**Figure** **3**; Figure S6, Supporting Information). These attached microbes may contribute to direct EET to the electrode surface, while cells in solution are not able to contribute the direct electron transfer.

Both the flavin‐secreting strains show higher currents compared to the empty vector control, with the rib‐dF strain achieving a maximum ≈3.4‐fold increase over the empty control (Figure S5a, Supporting Information). The relative contributions of the Mtr pathway and flavin pathway in the rib‐dF strain to the total current were assessed by comparing the current production from the strain harboring only the Mtr pathway (cymstcmtr) with the strain harboring only the corresponding rib‐dF flavin secretion pathway (rib‐dF‐dCyt) (**Figure** **4**a). Surprisingly, the exclusive expression of the rib‐dF flavin pathway leads to currents that are on par with those from the exclusive expression of the Mtr pathway. A comparison of the viability of the cells, however, shows a ≈28% lower optical density (OD_600_) for the rib‐dF‐dCyt strain compared to the cymstcmtr strain (Figure 4b). This decrease is observed for all flavin‐secreting strains (Figure 4b; Figure S5, Supporting Information), indicating that the overexpression of flavin biosynthesis genes negatively impacts strain viability. Accordingly, the EET enhancement is greater for the rib‐dF‐dCyt strain compared to the cymstcmtr strain on a per cell basis under the tested expression conditions. Accounting for baseline contributions from the control strain in the absence of both pathways (empty) and the ≈28% decrease in cell viability for the rib‐dF and rib‐dF‐dCyt strains compared to the cymstcmtr and negative control strains, the rib‐dF pathway demonstrates a ≈38% higher relative contribution per cell at the given levels of co‐expression. Though the current production from the rib‐dF strain appears largely as an additive combination of the increased current from the cymstcmtr strain and the increased current from the rib‐dF‐dCyt strain (see calculations in Supporting Information), the enhancement may underestimate the efficacy of the pathways, since the cytochrome expression levels are slightly lower when flavins are co‐expressed in the rib‐dF strain (Figure 2d; Figure S4, Supporting Information). Furthermore, in addition to the direct EET from the cells immobilized on the electrode, the Mtr pathway can also improve indirect EET through the reduction of diffusable, endogenous mediators.^[^ ^38^ ^]^ The Mtr‐expressing cells immobilized on the electrode may therefore contribute to both direct and indirect EET. The collective contributions from these cells, along with the indirect EET from the solubilized cells, limit the ability to discern the relative contributions of direct and indirect EET to the total current. The convolutive contributions from the Mtr pathway and the biosynthesis of the flavin therefore may not directly represent the contributions to direct and indirect EET, respectively.

## 3 Conclusion

Flavin overexpression is studied in *E. coli* cells in the presence and absence of the bioengineered Mtr pathway that has been previously developed for direct EET.^[^ ^15^ ^]^ The two newly modified strains rely on the overexpression of a riboflavin synthesis pathway, differing only in the lack or presence of *ribF*, a gene encoding the enzyme responsible for the conversion of riboflavin to FMN. The cytochromes of the Mtr pathway are expressed and correctly localized across strains, in agreement with the previous Mtr study that was performed in the absence of flavin overexpression.^[^ ^15^ ^]^ Although both modified strains show higher secretion of FMN compared to the previous Mtr strain, the strain containing *ribF* (rib) shows the highest total flavin secretion in addition to a higher FMN to riboflavin secretion ratio.

Given the role of flavins as redox mediators in *S. oneidensis* MR‐1^[^ ^12^ ^]^ and findings from previous efforts to further increase its flavin secretion,^[^ ^29^ ^]^ the increased flavin synthesis in *E. coli* is expected to analogously enhance the EET of the Mtr‐expressing strain. Consistent with these expectations, the chronoamperometry measurements confirm higher current for the flavin‐secreting strains, with the strain lacking the *ribF* (ribF‐dF) showing the highest EET, achieving a ≈3.4‐fold current increase over the control strain (empty). Since an increase in current is also observed for the previous Mtr‐expressing strain in the absence of flavin synthesis and the flavin‐synthesis strain in the absence of the Mtr expression relative to the control strain, these findings suggest that the Mtr and flavin secretion pathways contribute additively to enhancing the total EET in the combined strain. They further suggest riboflavin to be a more effective redox mediator than FMN, with the ribf‐dF strain achieving a higher current density despite its lower total flavin secretion compared to the FMN‐favoring rib strain.

Proposed future studies should focus on achieving greater control of the protein expression, mediator secretion, electrode engineering, and immobilization of the cells.^[^ ^24^, ^26^, ^39^, ^40^, ^41^, ^42^ ^]^ In this study, the flavins are studied under aerobic conditions, as no discernable flavin secretion was detected under anaerobic conditions. The ability to increase and control flavin secretion under anaerobic conditions is especially advantageous for anoxic applications, such as wastewater treatment using MFCs. Despite the overall improvement in EET, the flavin synthesis pathways appear to compromise cell viability, both when introduced in the presence and absence of the Mtr pathway. The engineered plasmid relies on a strong constitutive promoter for the steady‐state gene expression, which likely increases the metabolic stress of the cells. A range of promoters with different strengths could thus balance flavin secretion and viability to optimize EET. Such efforts for improving expression and secretion can be coupled with avenues for promoting biofilm formation. Since the C43(DE3) strains studied herein show limited biofilm formation,^[^ ^24^ ^]^ the EET is limited largely to suspended cells in solution. These cell suspensions enable clear differentiation of EET performance between the bioengineered strains in this study, without interference from secondary effects such as biofilm formation. In fact, previous studies have shown EET to be significantly enhanced through induced biofilm formation.^[^ ^39^ ^]^ The selection of biofilm‐forming strains or electrode configurations that facilitate cell immobilization may therefore further enhance charge extraction for improving current densities in the final application.

While the state‐of‐the‐art has largely focused on the exclusive expression of the Mtr pathway, the concomitant expression of flavin‐secretion pathways achieves a new benchmark for enhancing EET in *E. coli*. Beyond their complementarity for increasing EET, the cytochrome expression and flavin biosynthesis represent orthogonal pathways for EET. This orthogonality enables a new generation of multimodal microbial technologies, such as multiplexed sensing and chemical biosynthesis, that build on the electronic measurement and control of distinct metabolic pathways.

## 4 Experimental Section

### Molecular Cloning

Plasmids, primers, and strains used in this study can be found in the Supporting Information (Table S1). The empty vector pCDF‐empty was cloned using Gibson assembly.^[^ ^43^ ^]^ The backbone was amplified from pCDF‐mcherry1, and the ampicillin resistance gene was amplified from a pET vector using primers gibson_amppCDF_fwd, gibson_amppCDF_rev, gibson_ampR_fwd, and gibson_ampR_rev. The genes *ribA*, *ribB*, *ribDE*, and *ribF* were all amplified from an *E. coli* BL21 (DE3) genome extract using the corresponding primers. A total of six fragments were assembled using splicing by overlap extension PCR (primers SOE1_fwd, SOE1_rev, SOE2_fwd, SOE2_rev) in two separate reactions (SOE1: *ribDE*, *ribC*, *ribF*; SOE2: pCDF, *ribA*, *ribB*). The final products (SOE1 and SOE2) were joined using Gibson assembly to yield the plasmid pCDF‐ribABDECF. For cloning of the plasmid pCDF‐ribABDEC, pCDF‐ribABDECF was amplified using primers d_ribF_fwd and d_ribF_rev, followed by blunt‐end ligation using T4 DNA ligase. All cloning steps were performed using *E. coli* Dh5α, and all PCR reactions were performed using Q5 DNA polymerase (NEB).

### Cytochrome Expression

For cytochrome expression, three plasmids were co‐transformed into electrocompetent *E. coli* C43 (DE3) according to the combinations outlined in Table 1 to obtain the strains “empty”, “rib‐dF‐dCyt”, “cymstcmtr”, “rib”, and “rib‐dF”. For selection, kanamycin (50 µg mL^−1^), chloramphenicol (34 µg mL^−1^), and carbenicillin (100 µg mL^−1^) were used in both agar plates and liquid 2x YT medium. Overnight cultures were grown from single colonies in 5 mL 2x YT medium (15 mL tubes) at 37 °C and 220 rpm shaking. Expression cultures (25 mL 2xYT medium in 250 mL baffled Erlenmeyer flasks, 220 rpm shaking, supplemented with 1 mM 5‐aminolevulinic acid) were inoculated to an OD_600_ of 0.1 from overnight cultures, grown to an OD_600_ of ≈0.6 at 37 °C, induced with 10 µM IPTG, and grown overnight at 30 °C for 16 h.

### Subcellular Fractionation

Subcellular fractionation was carried out as previously described.^[^ ^15^ ^]^ For recovery of the periplasmic fractions, the cells were centrifuged, washed in phosphate buffer saline (PBS), resuspended in buffer A (100 mM Tris‐HCl pH 8, 500 mM sucrose, 0.5 mM EDTA), incubated for 5 min on ice, recentrifuged, and resuspended (gently using an inoculation loop) in 1 mM MgCl_2_ before incubating for 2 min on ice. After a final centrifugation step, the supernatants were collected as the periplasmic fractions and concentrated using 3 kDa molecular weight cut‐off Amicon filters. All centrifugation steps for the recovery of periplasmic proteins were carried out at 4 °C and 3000 g for 15 min each.

The pellets were collected for the recovery of membrane proteins. The cells were washed in buffer B (50 mM Tris‐HCl pH 8, 250 mM sucrose, 10 mM MgSO_4_), resuspended in buffer C (50 mM Tris‐HCl pH 8, 2.5 mM EDTA), and lysed using sonication. The lysates were clarified by centrifugation (10 000 g, 4 A°C, 10 min), and the membrane proteins were pelleted from the clarified lysates by centrifugation at 21 000 g for 4 h at 4 °C. The membrane pellets were washed once in buffer C, recentrifuged, and resuspended in buffer C supplemented with 1% Triton X‐100.

### SDS‐PAGE and Cytochrome Staining

Protein samples were separated at 150 V in 4%–20% SurePage (Genscript) polyacrylamide gels using MOPS‐SDS running buffer and 1x NuPAGE LDS sample buffer. After electrophoresis, the gels were rinsed in water, followed by light drying with a tissue to remove excess water. After application of an ECL stain (SuperSignal West Pico PLUS, Thermo Scientific) and incubation for 5 min, the gels were imaged using an ECL imager (Fusion Solo S, Vilber).

### Electrochemical Setup and Chronoamperometry

Chronoamperometry was carried out in a single‐chamber, 3‐electrode setup with an applied potential of +0.2 V vs. Ag/AgCl. The working electrode was composed of a platinum wire connected to a 1 × 1.1 × 0.5 cm piece of graphite felt (Alfa Aesar), and a platinum wire served as the counter electrode. The felt was pretreated by immersion in piranha solution (97% H_2_SO_4_:30% H_2_O_2_ in a 3:1 mixture) for 10 min, followed by washing and storage in distilled water. The chambers were filled with 95 mL M9 medium, prepared according to Baruch et. al.^[^ ^44^ ^]^ and supplemented with kanamycin (50 µg mL^−1^), IPTG (10 µM), and glucose (0.4%). Cytochrome expression cultures were washed (3 × 10 mL; 4 °C, 3000 g, 20 min centrifugation) and resuspended (5 mL) in the same medium before injection into the electrochemical setup during chronoamperometry (final volume of 100 mL) to a final OD_600_ of 0.6. Following chronoamperometry, the OD_600_ was measured to assess cell growth. A constant stream of N_2_(g) was run throughout the experiment to enable anaerobic measurements.

### HPLC Determination of Flavins

After cytochrome expression in 2x YT medium (see Cytochrome Expression), the cultures were centrifuged (4 °C, 3000 g, 20 min), and the pellets were washed in glucose‐supplemented M9 medium (see Electrochemical Setup and Chronoamperometry). After washing three times with 10 mL glucose‐supplemented M9 medium, the cells were used to inoculate 10 mL of M9 glucose medium in 100 mL Erlenmeyer flasks supplemented with kanamycin (50 µg mL^−1^), chloramphenicol (34 µg mL^−1^), and carbenicillin (100 µg mL^−1^). The cultures were kept at 30 °C and 220 rpm shaking, with 800 µL samples taken after 0, 6, 12, 24, and 48 h. For the anaerobic cultures, the same cells were used to inoculate 10 mL of glucose‐supplemented M9 medium supplemented with kanamycin (50 µg mL^−1^), chloramphenicol (34 µg mL^−1^), and carbenicillin (100 µg mL^−1^) in 12‐mL glass tubes sealed anaerobically using a rubber cap. The tubes were inoculated to an OD_600_ of 0.6 and kept for 96 h under magnetic stirring at room temperature.

Growth was monitored using OD_600_ measurements on a spectrophotometer (UV‐3600 Plus, Shimadzu). Filtered samples (0.2‐µm syringe filters) were used for HPLC measurements (Agilent Infinity II). The samples were separated on a C18 column (Agilent ZORBAX Eclipse Plus C18, RRHD, 2.1 × 50 mm, 1.8 µm) using ammonium acetate buffer (pH 6) and methanol as the mobile phase (0.9 mL min^−1^ flow rate, 30 °C). The methanol concentration in the mobile phase was kept at 8% for 1 min, increased to 20% over 7 min, increased to 70% over 30 s, kept at 70% for 3 min, decreased to 8% over 30 s, and finally kept at 8% for the last 3 min (15 min total run‐time per sample). Absorbance was measured at 210, 270, 375, and 450 nm (Agilent 1260 DAD WR).

For more sensitive measurements, anaerobic culture samples were additionally analyzed using an HPLC‐MS based on a Waters Acquity I‐Class UPLC system (Waters Corporation, Milford, MA, USA) coupled with a Waters Vion IMS‐QTof Mass Spectrometer equipped with LockSpray (leucine‐enkephalin, 200 pg µL^−1^). The instrument was controlled with Waters UNIFI 1.9.4 software (3.1.0, Waters Corporation, Milford, MA, USA). The instrument was operated with positive polarity in sensitivity mode (33 000 FWHM at m/z 556.2766). Data were acquired in HDMSe mode with a scan time of 0.072 s. The recorded mass range was from 50 to 550 m/z for both low and high energy spectra. The collision energy was ramped from 20 to 40 V. The cone voltage was set to 30 V, the capillary voltage was set to 1.5 kV, and the source offset was set to 50 V. The source temperature was set to 120 °C, and the desolvation temperature was set to 500 °C. The cone gas flow rate was set to 50 L h,^−1^ and the desolvation gas flow rate was set to 1000 L h^−1^. Separation was performed on an ACQUITY UPLC BEH C18 1.7 µm column, 2.1 mm x 50 mm (Waters), heated to 40 °C. The mobile phase was maintained at a flow rate of 0.4 mL min^−1^ and consisted of water (A) and MeOH (B). The gradient program over a total run time of 5 min was as follows: 0–0.5 min, 1% B; 0.5–3 min, 1–99% B; 4‐4.1 min, 99–1% B; and 4.1–5 min, 1% B to re‐equilibrate the system to initial conditions. The sample manager system temperature was set to 15 °C. Samples were injected without further dilution. The injection volume was 5 µL.

### SEM Imaging

The electrodes were removed from the electrochemical setup after chronoamperometry measurements and rinsed three times with water. The SEM imaging was performed as previously described.^[^ ^45^ ^]^ In brief, cells were fixed in 2% paraformaldehyde and 0.2% glutaraldehyde in 0.1 m cacodylate buffer (pH 7.2) for 3 h. After fixation, the cells were washed with the same buffer and then dehydrated using dimethylformamide (DMF) at increasing concentrations (25%, 50%, 75%, and 90%). The samples were stored at −20 °C until imaging, which was performed using a Zeiss Merlin microscope equipped with an Oxford Instruments X‐max Extreme EDX detector and Aztec software.

## Conflict of Interest

The authors declare no conflict of interest.

## Supporting information



Supporting Information

## Data Availability

The data that support the findings of this study are available from the corresponding author upon reasonable request.
