# Supplementary material for: Flavin Biosynthesis Enhances Extracellular Electron Transfer in Bioengineered Escherichia coli
Source: Adv Sci (Weinh). 2025 Nov 1;13(2):2412230. doi: 10.1002/advs.202412230 (PMC12786374; doi:10.1002/advs.202412230)
Supplement: Supplementary file 1 — Supporting Information [file ADVS-13-2412230-s001.docx]

**Supplementary information**

Flavin biosynthesis enhances extracellular electron transfer in bioengineered *Escherichia coli*

Mohammed Mouhib, Melania Reggente, Hanxuan Wang, Charlotte Roullier and Ardemis A. Boghossian

**Table S1. Overview of bacterial strains, plasmids and primers used in this study.**

| **Strains, plasmids and primers** | **Characteristics** | **Source** |
| --- | --- | --- |
| **Strains** |  |  |
| *E. coli* DH5α |  |  |
| *E. coli* C43 (DE3) |  |  |
| **Plasmids** |  |  |
| pEC86 | CcmABCDEFGH expression, chloramphenicol resistance, tet promoter | [33] |
| pSB1ET2 | empty backbone for cytochrome expression, kanamycin resistance, T7 promoter | [16] |
| pMO2.1 | CymA, STC and MtrCAB expression, kanamycin resistance, T7 promoter | [15] |
| pCDF-mcherry | constitutive mcherry expression, spectinomycin resistance, proD promoter | *** |
| pCDF-empty | empty vector for flavin biosynthesis genes, ampicillin resistance, proD promoter | this study |
| pCDF-ribABDECF | ribABDECF encoding plasmid, ampicillin resistance, proD promoter | this study |
| pCDF-ribABDEC | ribABDEC encoding plasmid, ampicillin resistance, proD promoter | this study |
| **Primers (5`- 3`)** |  |  |
| gibson_ribpCDF_fwd | CCGCCCGCGAATTTTTTGGGCTAACAAAACCGGCTTAATAAGGACGAGCCTCAGACC |  |
| gibson_ribpCDF_rev | AGTTTGGCTTCTGCCACACGTTTAAGCTGCATCTAGTATTTCTCCTCTTTCTCTAGTAGC |  |
| gibson_ribA_fwd | GATCGCATGGTTGCTACTAGAGAAAGAGGAGAAATACTAGATGCAGCTTAAACGTGTGG |  |
| gibson_ribA_rev | GTAAAAAAACCTCACTGAAATTATGGTTACCAGAATCAGCAAGAGGGTTATTTGTTCAGC |  |
| gibson_ribB_fwd | TGGGCCATTTGCTGAACAAATAACCCTCTTGCTGATTCTGGTAACCATAATTTCAGTGAG |  |
| gibson_ribB_rev | CTCCAGGCGCGCGATCTCTTCGCCAAATTCTTTAAGCAGCGGTTTTCAGCTG |  |
| gibson_ribDE_fwd | CACATGAGCGTAAAGCCAGCTGAAAACCGCTGCTTAAAGAATTTGGCGAAGAGATCG |  |
| gibson_ribDE_rev | CAACTCCTGAAATCAGTTAAGACATTCTGTTCAGTTACTAATTTCAGGCCTTGATGG |  |
| gibson_ribC_fwd | TTGAAAGCCATCAAGGCCTGAAATTAGTAACTGAACAGAATGTCTTAACTGATTTCAGG |  |
| gibson_ribC_rev | TCTGGCTCAAAACAGTGAAAATCGTCCGAGTAGATTTCAGATCAGGCTTCTGTACC |  |
| gibson_ribF_fwd | ATCAACCAGGTACAGAAGCCTGATCTGAAATCTACTCGGACGATTTTCACTGTTTTGAG |  |
| gibson_ribF_rev | GTCAGGTATGATTTAAATGGTCTGAGGCTCGTCCTTATTAAGCCGGTTTTGTTAGCCCA |  |
| SOE1_fwd | CCGCCCGCGAATTTTTTGGGCTAAC |  |
| SOE1_rev | CTCCAGGCGCGCGATCTCTTCG |  |
| SOE2_fwd | CACATGAGCGTAAAGCCAGCTG |  |
| SOE2_rev | GTCAGGTATGATTTAAATGGTCTGAGGCTC |  |
| gibson_amppCDF_fwd | ACTCTTCCTTTTTCAATATTATTGAAGCATTTATCAGG |  |
| gibson_amppCDF_rev | GATAGGTGCCTCACTGATTAAGCATTGGTAATGTCTAACAATTCGTTCAAGCCGAG |  |
| gibson_ampR_fwd | TCGGCTTGAACGAATTGTTAGACATTACCAATGCTTAATCAGTGAGGCAC |  |
| gibson_ampR_rev | GATAAATGCTTCAATAATATTGAAAAAGGAAGAGTATGAG |  |
| d_ribF_fwd | TAA GGA CGA GCC TCA GAC C |  |
| d_ribF_rev | AGA TCA GGC TTC TGT ACC TGG |  |

***pCDF-mcherry1 was a gift from Michael Lynch (Addgene plasmid # 87144 ; http://n2t.net/addgene:87144 ; RRID:Addgene_87144)


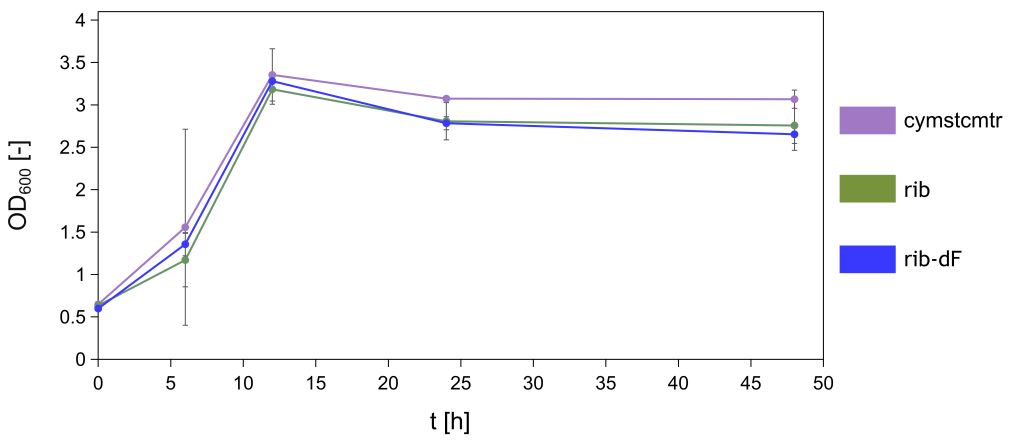


**Figure S1.** Change in OD_600_ over time during aerobic growth in glucose-supplemented M9 medium, with a starting OD_600_ of 0.6.


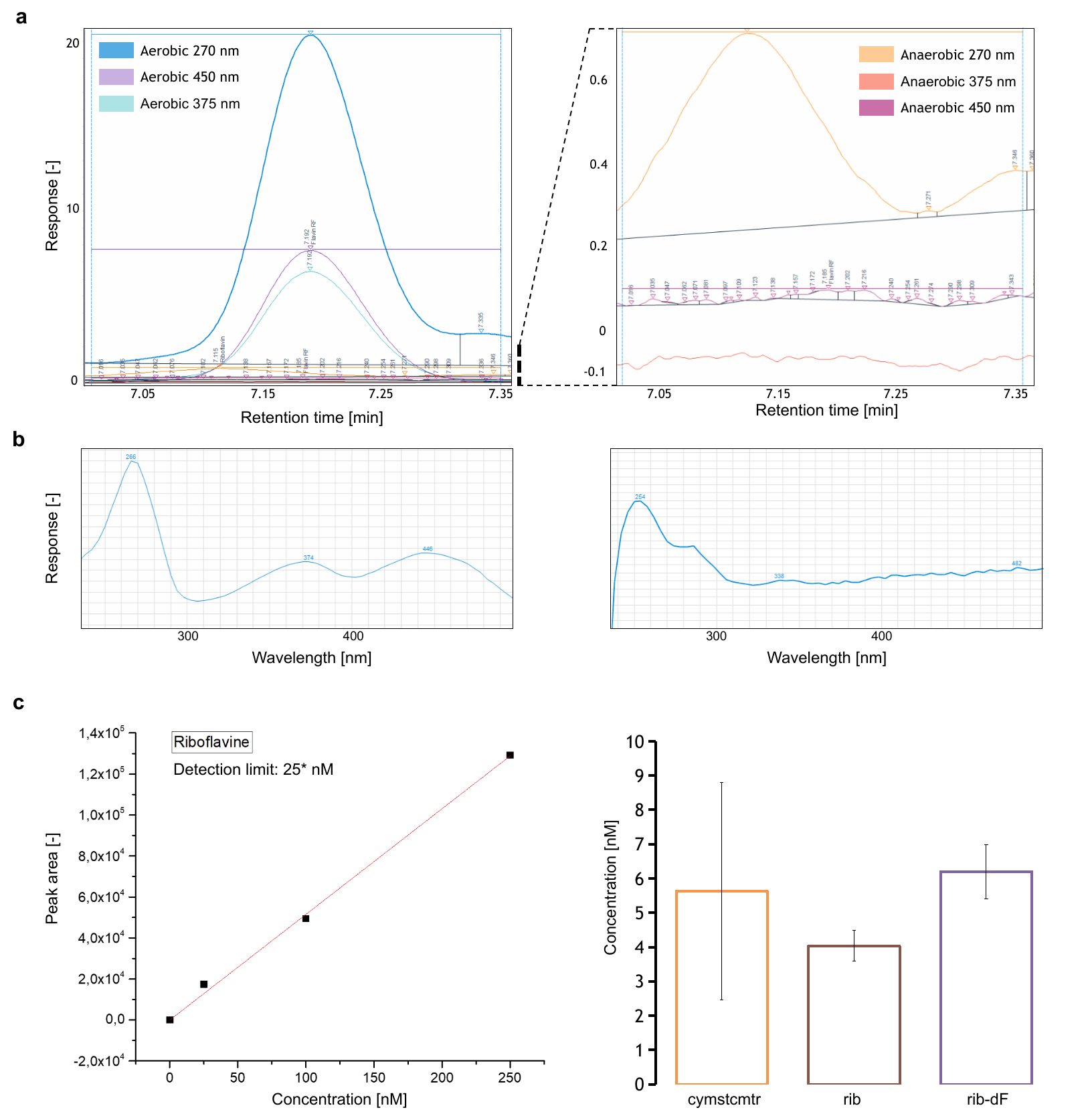


**Figure S2.** (a) HPLC measurements of culture samples (rib strain) to detect riboflavin biosynthesis after 96 h of aerobic (left) and anaerobic (right) growth. (b) Absorbance spectra of peak fractions at the expected riboflavin retention time for HPLC measurements above. (c) Riboflavin calibration curve and concentrations in culture samples from 96 h of anaerobic growth detected using an HPLC-MS method with higher sensitivity (25 nM detection limit).


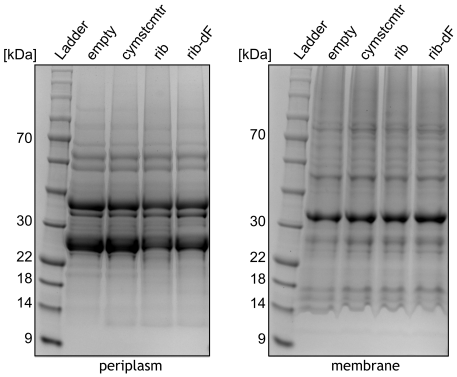


**Figure S3. Total protein staining of SDS-PAGE gels.** Gels were stained using Coomassie brilliant blue G250 following prior ECL staining of the same gels (see Figure 2d).


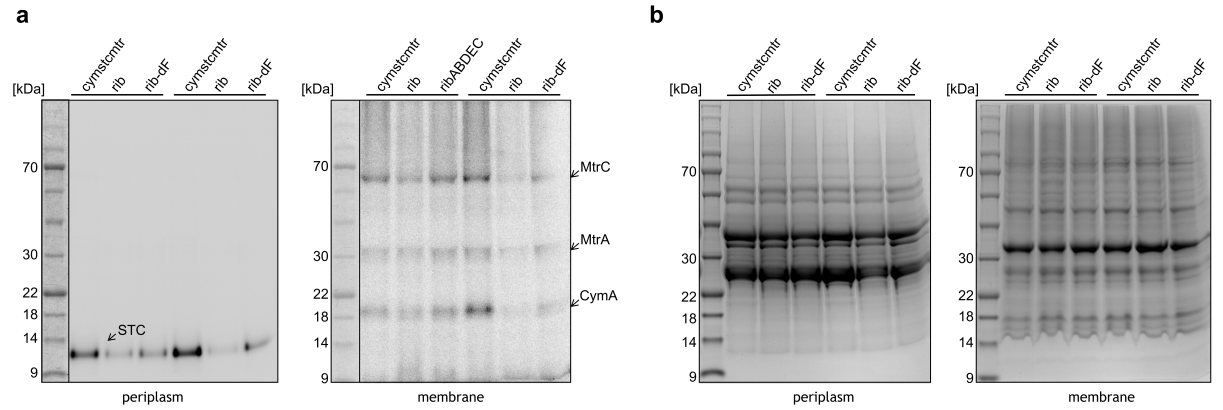


**Figure S4. Additional replicates to assess cytochrome expression and localization.** SDS-PAGE gels (4%-20%, MOPS-SDS buffer, 30 µg of protein per lane) loaded with periplasmic and membrane protein extracts were stained for (a) hemes using an enhanced chemiluminescence substrate and (b) total protein using Coomassie brilliant blue G250.


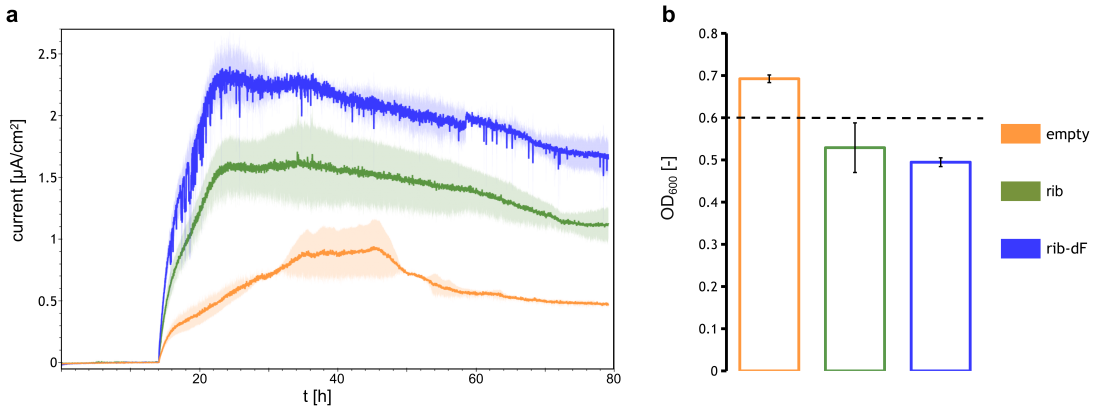


**Figure S5.** (a) Chronoamperometric measurements under anaerobic conditions in glucose-supplemented M9 medium. A potential of +0.2 V vs. Ag/AgCl was applied, and current values were averaged every 30 seconds. Mean currents of three independent measurements are plotted over time (two measurements for the empty vector control), with the shaded area representing one standard deviation. (b) The OD_600_ in solution measured after chronoamperometry. The horizontal dotted black line corresponds to the starting OD_600_ of 0.6. Bars represent mean values of three measurements, with the error bars corresponding to one standard deviation.



























**Figure S6.** SEM micrographs of a graphite felt working electrode (left) after chronoamperometry measurements with *E. coli* and (right) of a blank graphite felt sample. Images are shown at a 5000x, 10000x and 30000x magnification.

**Calculation of Relative Contributions of Mtr and Rib-dF Pathways to Total Current**

Values are approximated from Figure 3a, at time = 80 h:

j_Baseline_ = Baseline current density from empty vector strain (empty) ~ 0.5 μA/cm^2^

j_Mtr_ = Current density from Mtr pathway only (cymstcmtr) ~ 1.2 μA/cm^2^

j_Flavin_ = Current density from rib-dF flavin pathway only (rib-dF-dCyt) ~ 1.2 μA/cm^2^

j_rib-dF_ = Current density from Mtr + rib-dF pathways (rib-dF) ~ 1.8 μA/cm^2^

Taking into account that flavin-secreting strains (rib-dF-dCyt and rib-dF) show 28% lower viability compared to empty and cymstcmtr strains, predicted additive contributions from Mtr and rib-dF accounting for viability:

j_Predicted(rib-dF)_ = Baseline + Increase from Mtr Pathway + Increase from Flavin pathway

= 0.72*j_Baseline_ + (0.72*j_Mtr_ – 0.72*j_Baseline_) + (j_Flavin_ – 0.72*j_Baseline_)

= 0.72*0.5 μA/cm^2^ + (0.72*1.2 μA/cm^2^ – 0.72*0.5 μA/cm^2^) + (1.2 μA/cm^2^ – 0.72*0.5 μA/cm^2^)

= 1.7 μA/cm^2^ ~ 1.8 μA/cm^2^

The relative contribution of the rib-dF and Mtr pathways is approximated on a per cell basis:

j_Mtr_perCell_ = j_Mtr_/OD_600,Mtr_

j_Rib-dF_perCell_ = j_Flavin_/OD_600,rib-dF_

Because j_Mtr_ ~ j_Flavin_ and OD_600,rib-dF_ ~ 0.72*OD_600,Mtr_

j_Mtr_perCell_ ~ j_Rib-dF_perCell_ * OD_600,rib-dF_ /OD_600,Mtr_ = j_rib-dF_perCell_ * 0.72

or

j_rib-dF_perCell_ = 1.38*j_Mtr_perCell_
